# Probing Internalization Effects and Biocompatibility of Ultrasmall Zirconium Metal-Organic Frameworks UiO-66 NP in U251 Glioblastoma Cancer Cells

**DOI:** 10.3390/nano8110867

**Published:** 2018-10-23

**Authors:** Cataldo Arcuri, Lorenzo Monarca, Francesco Ragonese, Carmen Mecca, Stefano Bruscoli, Stefano Giovagnoli, Rosario Donato, Oxana Bereshchenko, Bernard Fioretti, Ferdinando Costantino

**Affiliations:** 1 Department of Experimental Medicine, Perugia Medical School, University of Perugia, Piazza Lucio Severi 1, 06132 Perugia, Italy; carmen.mecca@unipg.it (C.M.); rosario.donato@unipg.it (R.D.); 2 Department of Chemistry, Biology and Biotechnologies, University of Perugia, Via Elce di Sotto 8, 06123 Perugia, Italy; lorenzomonarca.92@gmail.com (L.M.); francescoragonese85@gmail.com (F.R.); bernard.fioretti@unipg.it (B.F.); 3 Department of Medicine, Perugia Medical School, University of Perugia, Piazza Lucio Severi 1, 06132 Perugia, Italy; stefano.bruscoli@unipg.it (S.B.); oxana.bereshchenko@unipg.it (O.B.); 4 Department of Pharmaceutical Sciences, University of Perugia, Via A. Fabretti 48, 06123, Perugia, Italy; stefano.giovagnoli@unipg.it

**Keywords:** UiO-66, nanoparticles, glioblastoma, biocompatibility, drug delivery

## Abstract

The synthesis of ultrasmall UiO-66 nanoparticles (NPs) with an average size of 25 nm, determined by X-ray powder diffraction and electron microscopies analysis, is reported. The NPs were stabilized in water by dialyzing the NP from the DMF used for the synthesis. DLS measurements confirmed the presence of particles of 100 nm, which are spherical aggregates of smaller particles of 20–30 nm size. The NP have a BET surface area of 700 m^2^/g with an external surface area of 300 m^2^/g. UiO-66_N (UiO-66 nanoparticles) were loaded with acridine orange as fluorescent probe. UV-vis spectroscopy analysis revealed no acridine loss after 48 h of agitation in simulated body fluid. The biocompatibility of UiO-66_N was evaluated in human glioblastoma (GBM) cell line U251, the most malignant (IV grade of WHO classification) among brain tumors. In U251 cells, UiO-66_N are inert since they do not alter the cell cycle, the viability, migration properties, and the expression of kinases involved in cancer cell growth. The internalization process was evident after a few hours of incubation. After 24 h, UiO-66_N@Acr (UiO-66_N loaded with acridine orange) were detectable around the nuclei of the cells. These data suggest that small UiO-66 are biocompatible NP and could represent a potential carrier for drug delivery in glioblastoma therapies.

## 1. Introduction

In the recent past, nanomedicine has become an attractive approach for targeted drug delivery and for new therapeutic strategies able to overcome the traditional limitations due to toxicity, healthy tissue damage, or other undesired side effects of direct drug administration [1,2,3]. The synthesis and application of nano-objects to be employed for therapeutic, pharmacological and diagnostic purposes has been rapidly growing [4,5]. Metallic nanoparticles (NPs), (i.e., plasmonic gold nanoclusters, silver NP, ferrite or magnetite superparamagnetic NP) of very small size are currently used as theranostic agents in living cells for a large number of diseases [6,7,8]. Nanomaterials are able to offer an efficient drug delivery by means of cellular internalization after encapsulation or surface attachment of the drugs. The use of nanocarriers such as lyposomes and dendrimers to be used in physiological conditions for drug delivery is also of great interest [9]. Among the mentioned compounds commonly used for these purposes, there is a class of materials called metal-organic frameworks (MOF) which has already been used for several application as drug delivery carriers and for imaging in living cells [10,11,12,13]. MOF are inorganic–organic hybrid compounds with porous crystalline structure and they are constituted of polynuclear metal clusters (also called nodes) linked each other by organic ligands such as carboxylate, phosphonates, heterocycles, and so on. The regular arrangement of nodes and ligands designs porous cages, normally filled by solvent molecules that make these materials suitable for transportation of bioactive molecules to be released in living cells [14,15,16]. Therapeutic agents and drugs—such as antibacterial and antiviral [17], as well as anticancer drugs like cisplatin [18]—have been successfully included in MOF NP and tested in living cells. In light of their low cytotoxicity, Zn and Fe based MOF are still the most employed. However, Zr based MOF are today considered the benchmark MOF materials in many fields [19]. UiO-66, which structure was first published by Lillerud et al. in 2008, has formula ((Zr_6_O_4_(OH)_4_(O_2_C−C_6_H_4_−CO_2_)_6_) and it is composed of hexa-zirconium(IV) oxo hydroxyl clusters, which are 12-connected by means of linear terephthalate linkers to form a cubic network with small tetrahedral and large octahedral cavities connected through narrow triangular pore windows about 1 nm wide [20]. It is well known that this structure is highly defective and the internal terephtalate linker can be replaced by monocarboxylic groups like acetate, benzoate, and formiate, thus increasing the internal pore volume [21]. Depending on the crystallinity degree the internal surface area can vary from 900 to 1300 m^2^/g and the pore volume from 0.35 to 0.5 cm^3^/g. Generally, UiO-66 crystals with 200 to 500 nm size can be obtained by conventional hydrothermal synthesis. However, crystals of ultrasmall size of 20–30 nm can be fabricated by properly changing synthetic conditions (amount of water, crystallinity modulator, and aging of the Zr(IV) solutions) [22,23]. Despite the ultrasmall size, the structure of UiO-66 is preserved at the expenses of a reduction of the internal surface area (400 m^2^/g, pore volume 0.16 cm^3^/g). On the other hand, the small size of the NP strongly increases the external surface area of about 5 to 10 times with respect to large crystals (up to 400 m^2^/g for 15 nm average size crystals) [23]. The use of such a small particles could be of potential interest for their potential capacity to easily pass the blood-brain barrier. Recently, surface modified UiO-66 nanocrystals have been used as luminescent sensors for cysteine and GHS detection [24] to unveil the endocytosis mechanism in He-La cells [25], for pH responsive drug delivery after surface PEGylation [26], as anticancer drug carriers after modification with ε-polycaprolactone [17], folic acid, and fluorescent markers (BODYPI) for enhanced cellular uptake [27]. UiO-66 NP have been recently employed after mechanical amorphization for studying the release of fluorescent dye calcein and α-cyano-4-hydroxycinnamic acid (α-CHC) [28,29] and also for miRNA detection [30]. Herein, we report the synthesis of ultrasmall UiO-66_N, their stabilization in water dispersion through dialysis, the loading with fluorescent acridine orange and the study of internalization and biocompatibility on U251 Glioblastoma cells line. Glioblastoma multiforme (GBM) is the most malignant (IV grade of WHO classification) and the most frequent among brain tumors of neuroepithelial origin [31]. The incidence in the United States is 2.96 cases/1,000,000 population/year with a higher peak in males older than 40 years of age [32]. The therapeutic approach to this tumor is complicated primarily by the proximity to the brain parenchyma, but also by the high infiltration capacity and low radio-sensitivity. Standard treatments include surgery, whenever possible, chemotherapy and radiotherapy, according to the Stupp’s protocol [33]. Nonetheless, the median survival remains 6 months after surgery alone, while surgery plus radiotherapy extends median survival to 12 months [34]. Moreover, recent experimental evidences have shown that, like other tumors, also GBM harbor a subpopulation of cancer stem cells, namely glioblastoma stem cells (GSCs) that are quiescent and thus evade radio and chemotherapy, causing tumor relapse [35]. To date, numerous genetic alterations of oncogene or tumor suppressor genes have been identified in GBM, including EGFR, PDGFRA, PIK3C2B, p16INK4a/p14ARF, PTEN, and RB1 [36], considering that many of these mutations result in an uncontrolled activation of tyrosine kinase receptors (TKR) and their downstream pathways, many efforts have been made to inhibit these deregulated pathways without significant result. The MOF incorporation in U251 cells was evaluated by flow cytometry after loading of the NP with acridine orange (UiO-66_N@Acr). Cytotoxicity studies, cell cycle evaluation, migration tests were also performed. Moreover, two major signaling pathways were also considered: ERK1/2 and PTEN/PI3K/Akt. The results obtained show that U251 cells internalize UiO-66_N without altering their physiology. These observations suggest that UiO-66_N may represent suitable nanocarriers to target drugs and/or active molecules to glioblastoma cells.

## 2. Materials and Methods

### 2.1. Chemicals

ZrCl_4_, acetic acid (99.5%), terephtalic acid, *N*,*N*-dimethylformamide were purchased from Sigma-Aldrich^®^ (St. Louis, MO, USA). The biological reagents are described along the experimental section.

### 2.2. Synthesis of Nanometric UiO-66_N (Average Crystal Size 25 nm)

UiO-66_N was prepared by dissolving 0.35 g of ZrCl_4_ (1.5 mmol) in 15 mL of DMF in a 50 mL Teflon vial. Then 0.635 mL of water (0.035 mmol) were added to the mixture. 1.4 mL (0.024 mmol) of acetic acid was added. The mixture was aged for 2 days at room temperature (RT). After that, 5 mL of 0.3 M 1,4 benzenedicarboxylic acid (BDC) solution in DMF was then added to the mixture. The Teflon vial was put in an oven at 120 °C for 24 h. After this time, a gel was recovered, washed twice with acetone and once with water. Then the solid was dried at 60 °C for two days and then soaked in chloroform.

### 2.3. Dialysis of UiO-66_N from DMF to Water.

In order to avoid the formation of big aggregates during the separation of the solid from DMF, UiO-66_N were purified by dialysis. Specifically, the reaction solution of UiO-66_N in DMF was withdrawn from the reaction vial and put in a dialysis tubing. The tubing was then closed at both sides and put into a beaker with deionized water, stirring for one week. The water in the beaker was changed every day. UiO-66_N dispersion appeared as a milky and homogeneous suspension that remains stable for week at RT. SEM analysis revealed that most of the UiO-66_N were aggregated in spherical clusters of 100–200 nm average diameter, as confirmed by DLS analysis.

### 2.4. Synthesis of Acridine Orange Loaded UiO-66_N (UiO-66_N@Acr)

50 mg of UiO-66_N (0.06 mmol) were first degassed overnight under vacuum and then dispersed in 5 mL of water. Then 5 mg of acridine orange (0.02 mmol) were added. The suspension was stirred at RT for three days. Then the solid was separated for centrifugation and washed three times with methanol. The amount of acridine orange absorbed by the MOF was evaluated from TGA analysis resulting in about 10 wt % of acridine orange absorbed from the MOF.

### 2.5. Gas Sorption Measurements

A Micromeritics 2010 apparatus (Micromeritics, Norcross, GA, USA) was used to obtain the adsorption and desorption isotherms with nitrogen at 77 K. Before the adsorption analysis the samples were first soaked in chloroform for two days. Then they were outgassed at 100 °C under vacuum overnight.

### 2.6. TGA Analysis

Thermogravimetric (TG) measurements were performed using a Netzsch STA490C thermoanalyser (NETZSCH Group, Selb, Germany) under a 20 mL min^−1^ air flux with a heating rate of 10 °C min^−1^.

### 2.7. Powder X-ray Diffraction

The PXRD patterns were collected in the 3°–60° 2θ range and with a 40 s/ step counting time with the CuKα radiation on a PANalytical X’PERT PRO diffractometer (Malvern Panalytical Ltd., Malvern, UK), PW3050 goniometer (Malvern Panalytical Ltd., Malvern, UK), equipped with an X’Celerator detector (Malvern Panalytical Ltd., Malvern, UK). The long fine focus (LFF) ceramic tube operated at 40 kV and 40 mA.

### 2.8. DLS and Zeta Potential

Size of UiO-66_N was investigated by dynamic light scattering (DLS) measurements in pure water at 20 °C. Briefly, dialyzed suspensions of UiO-66_N with or without acridine orange, prepared as reported above, were analyzed in ultrapure water at 20 °C. Analyses were performed using a Nicomp 380 ZLS photocorrelator (PSS, Santa Barbara, CA, USA) equipped with a 35 mWHe/Ne laser (λ = 658 nm) and an Avalanche photodiode detector. In the same conditions, zeta potential (ζ) was determined by measuring the electrophoretic mobility of particles at 20 °C. The scattering intensity was measured at 14° scattering angle over a time course of 180 s. The applied potential was 0.5 V cm^−1^.

### 2.9. Cell Culture Conditions

The U251 cell line was grown in DMEM with high glucose (EuroClone S.p.A., Milano, Italy) supplemented with 10% FBS (EuroClone S.p.A., Milano, Italy), 100 IU/mL penicillin G, 100 μg/mL streptomycin (EuroClone S.p.A., Milano, Italy) in an H_2_O-saturated 5% CO_2_ atmosphere at 37 °C.

### 2.10. Western Blotting

Cells were cultivated as detailed in the legends of the pertinent figure, washed twice with phosphate-buffered saline (PBS) and solubilized with 20% SDS, 1M Tris-HCl pH 7,4, 1M dithiothreitol, 200 mM PMSF, 10 mg/mL aprotinin (Gold Biotecnology, St. Louis, MO, USA), 1 mg/mL pepstatin (EuroClone S.p.A., Milano, Italy) and 5 mg/mL leupeptin (SERVA Electrophoresis GmbH, Heidelberg, Germany). Equal amounts of cell lysates were separated through 10% SDS page. The following antibodies were used: polyclonal anti phosphorylated (serine 473) Akt (1:1000), polyclonal anti Akt (1:1000), polyclonal anti phosphorylated (Thr202/Tyr204) Erk1/2 (1:1000), polyclonal anti total Erk1/2 (1:1000) (all from Cell Signaling Technology, Leiden, The Netherlands). The immune reaction was developed by SuperSignal West Pico Luminol/Enhancer Solution (Thermo Fisher Scientific, Waltham, MD, USA). Filters were subjected to densitometric analysis of the pertinent immune bands and their relative standard references using the software Image Studio Digit (LI-COR, Lincoln, NE, USA).

### 2.11. Measurement of Cell Cycle and Apoptosis

Cells were treated with Ethanol and UiO-66_N at 1 μg for 24 h and 48 h respectively; then the culture medium was collected and centrifuged (400× *g*, 7 min) in order to recover cells in suspension and cells were washed two times with PBS and processed for cell cycle analysis by propidium iodide staining and flow cytometry. Briefly, the cells were resuspended in 0.4 mL of hypotonic fluorochrome solution (50 μg/mL propidium iodide in 0.1% sodium citrate plus 0.1% Triton X-100) in 12 × 75-mm polypropylene tubes (BD Biosciences Italy, Milano, Italy). The tubes were kept at 4 °C for at least 30 min before flow cytometric analysis. The propidium iodide fluorescence of individual nuclei was measured using a FACScan flow cytometer (BD Biosciences Italy, Milano, Italy) at 488 nm. The percentages of cells in G0/G1, S, and G2/M phases and apoptotic cells were calculated using Cell FIT cell cycle analysis version 2.0.2 software.

### 2.12. Scratch/Wound Healing Assay

Cells were grown to confluent monolayer and, when the confluence reached the 100%, the surface was scratched as uniformly as possible with a pipette tip forming a wound. This initial scratch and the movement of the cells into the wound area were photographed using the Olympus IX51 microscope (Olympus, Tokyo, Japan) with a 4× magnification until the wound area of the control sample was definitively closed. The size of the wound’s area of all samples was calculated at each time point using the open source software ImageJ (National Institutes of Health, Bethesda, MD, USA). Two independent series of experiments were performed for each cell line.

### 2.13. Internalization of UiO-66_N by U251 Glioblastoma Cell Line

One hundred thousand cells were seeded into 35 mm Petri dish plates (Thermo Fisher Scientific, Waltham, MD, USA) in complete medium. After 24 h the medium was renewed and ethanol and UiO-66_N@Acr at the final concentration of 30 μg/mL were added. After 24 h and 48 h, the culture medium was discharged, cells washed five times with PBS, detached with trypsin/EDTA (0.1%), collected and centrifuged (400× *g*, 7 min). The cells were resuspended in 0.4 mL of PBS and tubes were kept at 4 °C for at least 30 min before flow cytometric analysis. The UiO-66_N@Acr fluorescence of individual cells was detected with Coulter Epics XL-MCL flow cytometer (Beckman Coulter, Brea, CA, USA) and data were analyzed using FlowJo software (TreeStar, Ashland, OR, USA).

### 2.14. MTT Viability Assay

Cells were seeded in 96 well plates with a cell density of 4 × 10^3^ and after 24 h in culture were treated with UiO-66_N in pure water at the final concentrations of 0.1, 0.5, 1, 5 and 10 μg/mL. 24 and 48 h later, cells were incubated with the 3-(4,5-Dimethylthiazol-2-yl)-2,5-Diphenyltetrazolium Bromide (MTT) solution (Sigma Aldrich®, St. Louis, MO, USA) for four hours and after this incubation period, a water-insoluble formazan dye was formed. After solubilisation, the formazan dye was quantified using a LabSystems Multiskan MS spectrophotometer at 550 nm (Artisan Technology Group ®, Champaign, IL, USA). Each experiment was performed in triplicate.

### 2.15. FURA-2 Calcium Imaging Assay

U251 cells were plated at the concentration of 1.5 × 10^3^ cells/mL and used on the third day of culture. Before experiments, cells were incubated with FURA-2 AM (3 μM; Sigma-Aldrich, Sigma Aldrich®, St. Louis, MO, USA) for 45 min and extensively washed with a Ringer solution of the following composition (in mM): NaCl 106.5, KCl 5, CaCl_2_ 2, MgCl_2_ 2, MOPS 5, glucose 20, Na gluconate 30, at pH 7.25 (all from Sigma Aldrich®, St. Louis, MO, USA). Cells were continuously perfused using a gravity-drive perfusion system, with tubing connected to a final tip of 100 to 200-μm diameter focally oriented onto the field of interest. UiO-66_N were added at the concentration of 1 μg/mL after 9 min of perfusion for 8 min. A standard control pressure perfused the cells for about 20 min with only the Ringer solution. A positive control pressure perfused a solution of ionomycin calcium salt (Tocris Bioscience, Bristol, UK) for about 7 min. The estimation of intracellular free Ca^2+^ concentration was reported as change of the ratio between fluorescence emission at 510 nm, obtained with 340 and 380-nm excitation wavelengths (optical filters and dichroic beam splitter were from Lambda DG4, Shutter Instruments, Novato, CA, USA). Ratiometric data was randomly acquired from 30 cells every 3 s. 

### 2.16. Microscopic Fluorescence Observation and Analysis

All the imaging analyses have been conducted with an upright fluorescence microscope (Axiozoom V16, Zeiss, Oberkochen, Germania); all the images are snapped with a digital camera (Axiocam 502 mono, Zeiss, Oberkochen, Germania) and elaborated with ZEN 2 software (Zeiss, Oberkochen, Germania).

### 2.17. Statistical Analysis

Each experiment was performed at least three times and data are expressed as mean values ± SEM. Data were subjected variance (ANOVA) analysis using a statistical GraphPad Prism, version 7.00 software package (GraphPad Software, La Jolla, CA, USA).

## 3. Results

UiO-66_N was synthesized in a nanometric form according to a modified synthetic procedure [22]. This synthesis produced UiO-66_N nanocrystals in the low nanometric range with an average size around 25 nm. To the best of our knowledge, this is the first report on ultrasmall MOFs being investigated for their cell internalization capacity.

**Figure 1:**
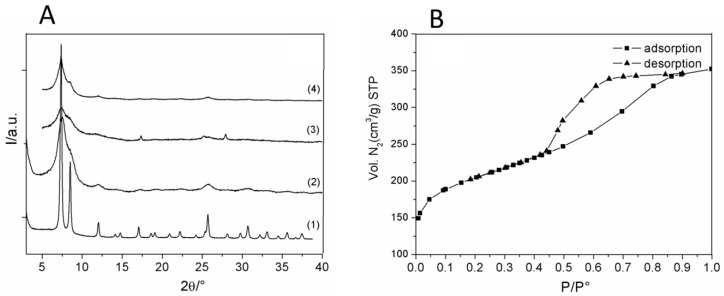
(**A**) XRPD patterns for UiO-66_N 100 (1), UiO-66_N (2), UiO-66_N soaked overnight in chloroform (3), and UiO-66_N@Acr (4); (**B**) N_2_ adsorption-desorption isotherms for sample UiO-66_N measured at 100 K.

Figure 1A displays the XRPD patterns of UiO-66_N with 100 nm average particle size (1); showed for comparison, of UiO-66_N (25 nm average particle size) (2); UiO-66_N soaked overnight in chloroform (3); and UiO-66_N loaded with acridine-orange (4). The average crystal size was estimated by applying the Scherrer formula on the 111, 200 and 220 peaks after analytical deconvolution and by correcting the instrumental broadening with a line profile standard (LaB_6_), according to a procedure already applied for similar materials [36]. For the nanometric samples the average crystalline domain size obtained, after the application of the Scherrer formula, was 15 nm. A drastic broadening of the peaks, in agreement with very small crystalline domains, was indeed observed. From the comparison of patterns (1) and (2), it can be seen that the structure of UiO-66-N is fully retained although an important peak broadening is observed. The soaking in chloroform was used in order to remove the residual DMF solvent molecules, the successive acridine inclusion did not change the X-ray pattern. The amount of acridine loaded is about 10 wt % as calculated from the TGA analysis (Figure S1). The nitrogen adsorption isotherm of UiO-66_N is shown in Figure 1B and the BET surface area is 736 m^2^/g. The hysteresis of the desorption curve is indicative of the presence of mesopores probably due to interparticle contacts. The micropore surface area is 411 m^2^/g (pore volume of 0.17 cm^3^/g), whereas the external surface area is about 325 m^2^/g. These results are in good agreement with those recently reported on UiO-66 nanocrystals with size around 20 nm [22,23]. The value of the external surface area is about 5 to 10 times higher than that normally measured for UiO-66_N samples with average crystalline size > 100 nm. However, an important reduction of the internal surface area (more than a half) compared to that normally measured for crystalline UiO-66_N (around 1000 m^2^/g) was observed.

**Figure 2:**
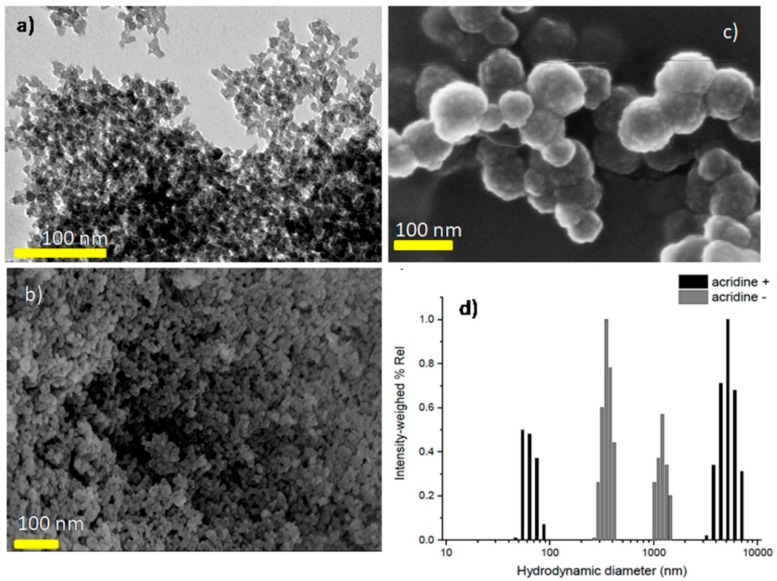
(**a**) TEM and (**b**) FE-SEM images of UiO-66_N obtained by hydrothermal synthesis; (**c**) spherical particles aggregates of smaller UiO-66_N@Acr obtained by dialysis; and (**d**) photo correlation spectroscopy measurements depicting hydrodynamic populations for UiO-66_N (grey) and UiO-66_N@Acr (black) in pure water at 20 °C.

Figure 2a,b show the TEM and FE-SEM images of UiO-66_N obtained by direct synthesis. They appear to be strongly aggregated but their size has a homogeneous distribution centered around 30 nm, which is a value slightly higher than that found by XRPD data (see Figure S2). However, the broadening of the X-ray peaks also takes into account the presence of defects resulting in a larger broadening than that only related to the crystal size. Another SEM image of UiO-66N is placed in Supporting Information (Figure S7) Figure 2c shows the UiO-66_N separated from DMF by dialysis and loaded with acridine. The picture was taken from the sample dried from the water dispersion after dialysis. The small particles are aggregated into spherical structures of 100 to 200 nm size. These spherical structures are also aggregated in larger clusters being attached each other. Photo correlation spectroscopy (Figure 2d) confirmed the observed formation of large aggregates especially for UiO-66_N@Acr. The dialyzed suspensions showed populations with mean hydrodynamic diameters of 65 nm and 345 nm with large aggregates at size > 5000 nm and 1000 nm for UiO-66_N@Acr and UiO-66_N, respectively. Such findings clearly highlight once more the tendency of UiO-66_N to aggregate in water, matching the TEM observations on the non-dialyzed sample. Even though more dispersed than UiO-66_N, the presence of a smaller particle population for UiO-66_N@Acr particles may suggest that a change in surface properties due to acridine orange adsorption could prevent to some extent aggregation. Such a modification is proved by the change measured in the ξ value that changed from negative (−14.8 mV) for UiO-66_N to slightly positive (+4.1 mV) for UiO-66_N@acr as a result of the presence of acridine on the surface of the particles. Therefore, acridine may produce some small repulsion able to partially reduce particle agglomeration.

**Figure 3:**
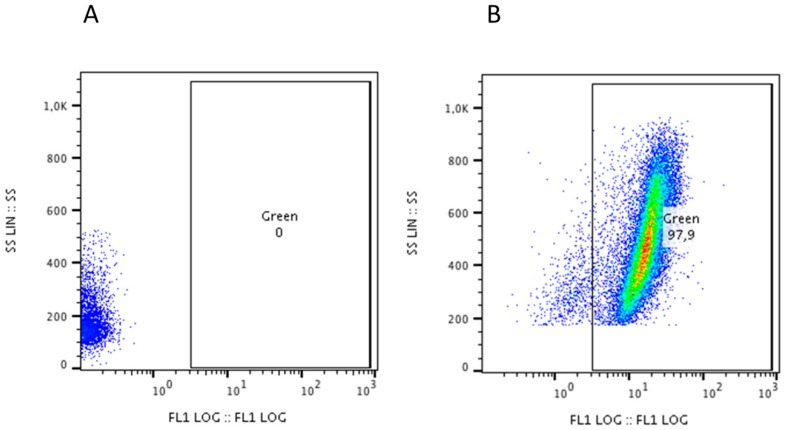
UiO-66_N internalization by U251 cells. (**A**) Not treated cells showed no fluorescence. (**B**) UiO-66_N@Acr treated cells showed fluorescence after 48 h of treatment. Fluorescent cells accounts for 97.9% of total cells.

In order to check the acridine adsorption/inclusion degree inside the MOF structure, the effective release of such a dye in PBS (which simulate the cell culture conditions) was studied by UV-vis spectroscopy. UV-vis spectra of UiO-66_N@Acr dispersed in PBS (about 2 mg/mL) are shown in Figure S3. After 24 h and 48 h, no acridine absorption signal could be detected suggesting that no molecules were released from the MOFs. This experimental evidence excludes the presence of free acridine molecules into the cells over the entire incubation timeframe. As our main interest is directed toward pharmaceutical applications, the biological inertness of our MOFs must be a pivotal feature. Since no acridine molecules are released from the MOF, we have evaluated the UiO-66_N@Acr incorporation in U251 cells. After 48 h of 30 µg/mL UiO-66_N@Acr treatment, the 97.9% of the cells showed fluorescence assessed by cytofluorimetry (Figure 3B) compared to untreated cells (Figure 3A) that did not show fluorescence. The same percentage of U251 fluorescent cells was shown after 24 and 72 h of treatment (data not shown). These results demonstrate that UiO-66_N are quickly incorporated by U251 cells and held permanently inside them for at least 72 h.

**Figure 4:**
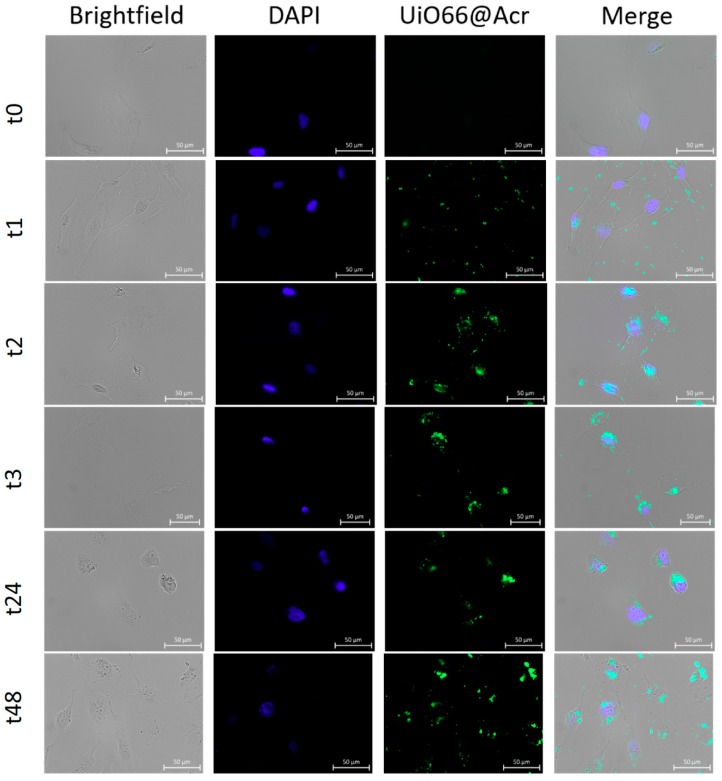
Fluorescence imaging of cells treated with UiO-66_N@Acr at the final concentration of 1 μg/mL at different times (0, 1, 2, 3, 24, and 48 h) of treatment. Bar 50 μm

In order to confirm UiO-66_N internalization, U251 cells were treated with UiO-66_N@Acr at the final concentration of 1 μg/mL for 48 h and observed by immunofluorescence. Before observation, excess of MOFs in the cell medium was removed by extensive washes in PBS; nuclei of cells have been marked with DAPI. Cells were observed alive in PBS. Results of fluorescence imaging are shown in Figure 4: UiO-66_N@Acr are detectable inside U251 glioblastoma cells after 2 h from the incubation and they are still detectable after 48 h. The accumulation of MOFs seems to concentrate around the nuclei of the cells (Figure 4).

**Figure 5:**
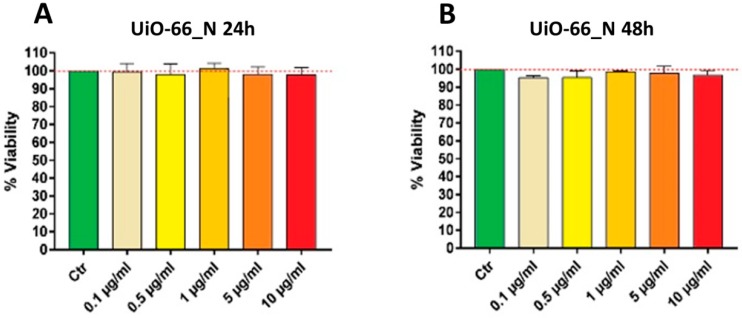
U251 cells viability by MTT assay, after 24 (**A**) and 48 (**B**) hours of incubation with UiO-66_N at various concentrations.

Cells have been washed with PBS and incubated with DAPI for three minutes, in order to stain cellular nuclei. Cells have been washed again before observation. Figure 5 displays the biocompatibility of UiO-66_N in terms of viability: MTT assay shows no significant changes in U251 cells viability compared to control after 24 and 48 h of exposition at different concentrations (Figure 5). MTT assay uses tetrazolium salt 3-(4,5-dimethyl-2-thia-zolyl)-2,5-diphenyl-2H-tetrazolium bromide) as a biological tool to measure cell viability. Reduction of MTT is associated with flavin-containing enzymes, which are well-known mitochondrial enzymes, and this demonstrates that mitochondria are the main site of MTT reduction. Thus, UiO-66_N do not alter cell viability and do not interfere with the mitochondrial activity of the cells.

As calcium plays an important role in cell physiology, a ratiometric intracellular calcium concentration measurement with the FURA-2 assay following acute treatment with UiO-66_N was performed. Data shown in Figure S4, displays no significant changes in the intracellular calcium concentration following the application of 1 μg/mL on UiO-66_N; Similar results were obtained with the higher concentration of 10 μg/mL UiO-66_N (Figure S5). The FURA2 assay was conducted even without MOF perfusing, as an assay control showing no differences with the previously described experiment. Such data support further MOF biocompatibility.

**Figure 6:**
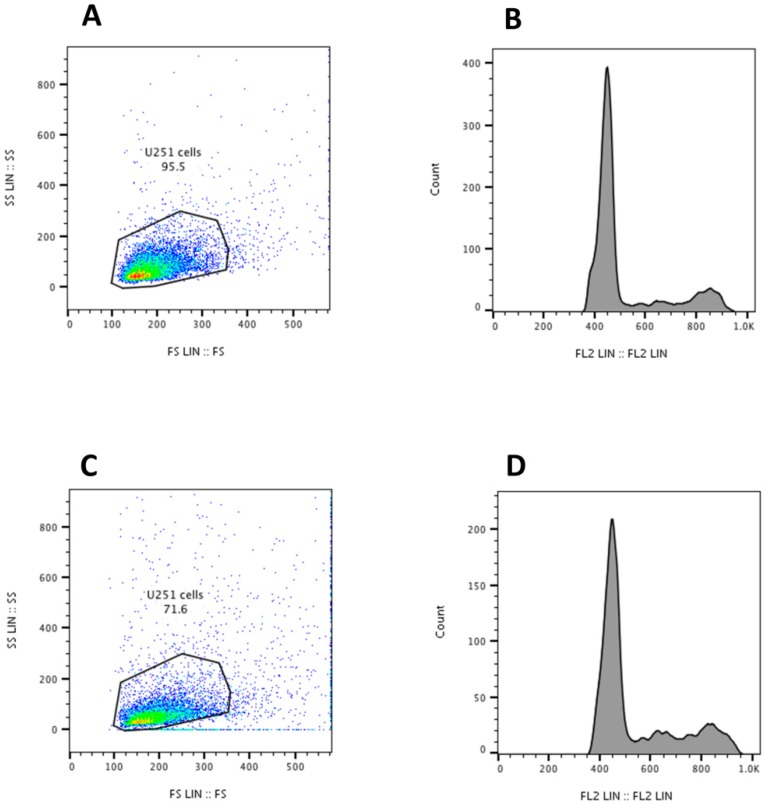
Flow cytometry analysis of not treated (**A**,**B**) or treated (**C**,**D**) U251 cells with UiO-66_N (30 µg/mL) for 48 h. Left panels represent dot plot of not treated (**A**) and UiO-66_N treated (**C**) live U251 cells (gated inside the dot plot). Events outside the gate of U251 cells in UiO-66_N-treated group are mostly MOF aggregates. Right panels represent propidium iodide staining showing cell cycle profiles. No significant differences between not treated (**B**) and UiO-66_N treated (**D**) cells were detected.

Having demonstrated that UiO-66_N are quickly incorporated, the cell cycle of U251 cells to exclude UiO-66_N effects on cell cycle machinery was evaluated. Propidium iodide staining shows no significant differences between not treated and MOF-treated cells (Figure 6), further confirming that they are a biocompatible and an inert nanomaterial. In accordance with these findings, the percentage of cell distribution in G0/G1, S, and G2/M phases was not modified between not treated and MOF-treated cells (data not shown).

**Figure 7:**
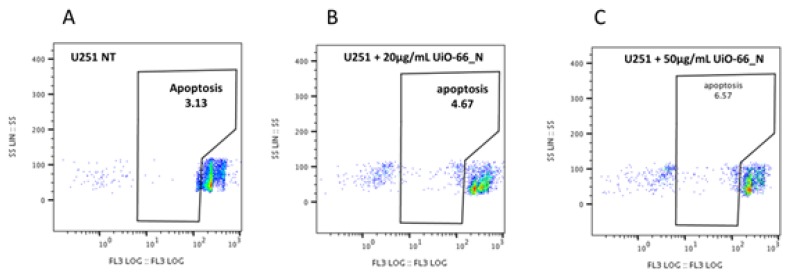
UiO-66_N administration does not induce apoptosis in U251 cells. Flow cytometry analysis of not treated (**A**) or treated U251 cells with 20 (**B**) or 50 (**C**) μg/mL UiO-66_N for 48 h. Numbers inside the gates represent percentage of apoptotic U251 cells assessed by propidium iodide staining.

Moreover, by FACS analysis, we measured the percentage of apoptotic cells in UiO-66_N (20 or 50 μg/mL) treated U251 cells compared to not treated cells (Figure 7). After 48 h of treatment, the percentage of apoptotic U251 cells in treated and not treated cells was similar, with slight not statistically significant differences. These results demonstrate that in U251 cells UiO-66_N does not affect apoptosis.

**Figure 8:**
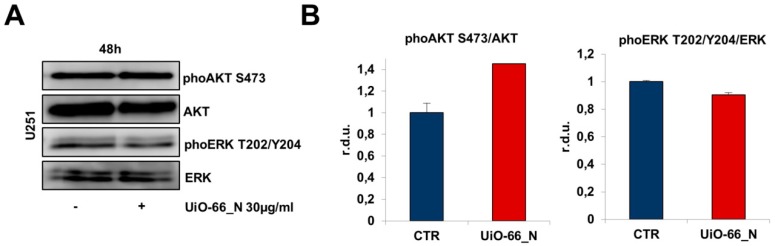
UiO-66_N do not affect Akt and ERK1/2 phosphorylation in U251 glioblastoma cells. Western blots (**A**) and densitometric quantification of Akt and ERK1/2 phosphorylation levels (**B**). The cells were treated with 30 µg/mL UiO-66_N for 48 h. Blots are representative of at least three experiments. The difference in phoAkt level is not statistically significant.

In GBM, high level of phosphorylated AKT has been reported to correlate with a poor prognosis [36]. Epidermal growth factor receptor (EGFR) amplification and/or overexpression occurs in 40–50 % of GBM [30] and leads to the activation of PI3K/AKT signaling pathway. [36]. Thus, inhibition of this pathway seems to be a promising target for developing more effective GBM therapies. However, the modest efficacy of these approaches observed in the clinical trials conducted so far, suggests that they might be more effective in combination with other agents. [37] In GBM, EGFR overexpression also activates the extracellular signal-regulated kinases 1 and 2 (ERK1/2), [32] proteins belonging to the mitogen-activated protein kinase (MAPK) family contributing to the highly altered phenotype of this tumor cells. UiO-66_N did not affect Akt and ERK1/2 phosphorylation level in U251 glioblastoma cells. From a metabolic point of view UiO-66_N showed a completely inert behavior (Figure 8).

Invasiveness is considered as a major determinant for malignant behavior in human gliomas and U251 cells represent a highly invasive tumor [38]. For this reason, the involvement of UiO-66_N in influencing migration properties was investigated. In order to avoid filling up of the wound by proliferating rather than migrating cells, these tests were conducted under non-proliferative conditions. As expected, UiO-66_N did not affect U251 cell migration evaluated by wound healing assay, compared to not treated cells (Figure S6). It is noteworthy that glioblastoma infiltration is an extremely complex phenomenon that also requires the steady support of extracellular cues [39]. Our preliminary data demonstrate that UiO-66_N do not affect this phenomenon.

## 4. Conclusions

UiO-66 nanocrystals of ultrasmall size were synthesized and tested for the first time on glioblastoma U251 cells in order to evaluate their biocompatibility and internalization mechanism. The UiO-66 N were used without any surface modification. The particles separated from the mother liquors tend to strongly aggregate and they cannot be easily stabilized in water dispersion. However, dialysis of the NPs from the DMF solution to water allows to obtain stable dispersion of 100 nm spherical aggregates. Despite the absence of surface modification, nude UiO-66_N are avidly internalized by GBM cells and retained within them. No morphological changes or decrease in cell viability were highlighted. Similarly, the cell cycle, the apoptosis as well as the migration capacity were not altered. From a metabolic point of view, neither changes in mitochondrial activity nor alterations of fundamental transduction pathways were evidenced. Ultimately UiO-66_N are ideal for drug and active molecule delivery towards GBM cells. The treatment of GBM represents a very complex challenge and new strategies must be put into place. Nanomedicine can provide new opportunity for this disease. To date, different NPs have been utilized for drug delivery in GBM, through different administration routes [40]. Various criticisms, such as restricted passage across the blood–brain barrier, have been highlighted. Moreover, GBM are characterized by high infiltrative ability and the potential therapeutics should not only be able to pass through the blood–brain barrier but also should be able to diffuse within the brain. In this context, future studies will have to address not only the brain accumulation and infiltration capacity of these NP but also strategies to grant selectivity with respect to GBM. If successful, ultrasmall UiO-66_N may lead to new treatment approaches for GBM treatment.

## Supplementary Materials

The following are available online at http://www.mdpi.com/2079-4991/8/11/867/s1, Figure S1: TGA curves of UiO-66_N (blue line) and UiO-66_N@Acr (orange line); Figure S2: Size distribution of UiO-66_N obtained by direct synthesis; Figure S3: UV-vis spectra in simulated body fluids (SBF) of acridine orange (orange line), UiO-66_N@Acr (red line) and SBF surnatant after 24 h and 48 h; Figure S4: Calcium channels activation in untreated cells; Figure S5: Calcium channels activation during treatment with UiO-66_N; Figure S6: Wound healing assay of U251 cells; Figure S7: High magnification FE-SEM image of UiO-66_N.
