# Supplementary material for: Probing Internalization Effects and Biocompatibility of Ultrasmall Zirconium Metal-Organic Frameworks UiO-66 NP in U251 Glioblastoma Cancer Cells
Source: Nanomaterials (Basel). 2018 Oct 23;8(11):867. doi: 10.3390/nano8110867 (PMC6267206; doi:10.3390/nano8110867)
Supplement: Supplementary file 1 [file nanomaterials-08-00867-s001.zip › nanomaterials-372179-supplementary.doc]

Supplementary Materials

# Probing Internalization Effects and Biocompatibility of Ultrasmall Zirconium Metal-Organic Frameworks UiO-66 NP in U251 Glioblastoma Cance Cells

Cataldo Arcuri^1^, Lorenzo Monarca^2^, Francesco Ragonese^2^, Carmen Mecca^1^, Stefano Bruscoli^3^, Stefano Giovagnoli^4^, Rosario Donato^1^, Oxana Bereshchenko^3^, Bernard Fioretti^2^ and Ferdinando Costantino^2^*

^1^ Department of Experimental Medicine, Perugia Medical School, University of Perugia, Piazza Lucio Severi 1, 06132 Perugia, Italy

^2^ Department of Chemistry, Biology and Biotechnology, University of Perugia, Via Elce di Sotto 8, 06123 Perugia, Italy

^3^ Department of Medicine, Perugia Medical School, University of Perugia, Piazza Lucio Severi 1, 06132 Perugia, Italy

^4^ Department of Pharmaceutical Sciences, University of Perugia, Via A. Fabretti 48, 06123, Perugia, Italy

**C**orrespondences: ferdinando.costantino@unipg.it (F.C.); cataldo.arcuri@unipg.it (C.A.);

**Figure S1.** TGA curves of UiO-66_N (blue line) and UiO-66_N@Acr (orange line). TGA analysis of UiO-66_N reveals a weight loss of about 28% measured from 300 °C which is normally related to dehydroxylation. The weight loss is sensibly lower than that calculated for a defect free Zr_6_O_6_(bdc)_6_ UiO-66 phase. It mights indicate that a high amount of monocarboxylic modulator used for the synthesis is present into the structure. In our case acetic acid was used. The NMR analysis of hydrolyzed MOF in NaOD 1M confirmed the presence of acetic acid (2.0 ppm, s, D_2_O) into the MOF structure meaning that the phase is highly defective.

**Figure S2.** Size distribution of UiO-66_N obtained by direct synthesis.

**Figure S3.** UV-Vis spectra in simulated body fluids (SBF) of acridine orange (orange line), UiO-66 _N@Acr (red line) and SBF surnatant after 24h and 48h.

**Figure S4.** Calcium channels activation in untreated cells. Perfusing solution: Ringer solution (1-2-3), Ionmycin 1 μM in Ringer solution (4).

**Figure S5.** Calcium channels activation during treatment with nano-UiO-66_N. Perfusing solution: Ringer solution (1), nano-UiO-66_N 1 μg/ml in Ringer solution (2), wash-up in Ringer solution (3), Ionmycin 1 μM in Ringer solution (4).

**Figure S6.** Wound healing assay of U251 cells. Cells were grown to confluence and scratched with a pipette tip to generate a wound. Immediately after the scratch , the culture medium was replaced with serum- free DMEM plus UiO-66_N (30µg/ml). The wound areas were photographed (A) and analysed (B) with Image J (MRI_wound_healing_tool-6), bar=100µm.

**Figure S7.** High magnification FE-SEM image of UiO-66_N .
